# Fatal attraction: Dengue Virus and the Mitochondrial Connection

**DOI:** 10.1080/27694127.2023.2167429

**Published:** 2023-01-19

**Authors:** Bharati Singh, Avula Kiran, Shamim A Sufi, Gulam Hussain Syed

**Affiliations:** Institute of Life Sciences, Bhubaneswar, Odisha, India

**Keywords:** Dengue, inflammation, mitochondria homeostasis, mitophagy, mt-DNA

## Abstract

Viruses rely on host cell machinery, an obligation that forces a myriad of interactions between the viral proteins and host cell organelles to create an intracellular environment conducive to viral dissemination. In their pursuit to exploit the cellular organelles and modulate their function, viruses affect the overall physiology of the cell. The consequence of viral infection on host cell physiology largely varies between acute *vs* chronic infection, cell type infected, and the productive or abortive nature of the infection. Viruses leading to chronic infection favor viral persistence by promoting the viability of infected cells by inducing cellular homeostatic pathways. In contrast, viruses causing an acute infection trigger physiological alterations that often lead to the cytocidal effect.

Mitochondria perform several vital functions and govern cellular bioenergetics, metabolism, calcium homeostasis, and anti-viral defense. Owing to their pleiotropic role, mitochondria are the primary cellular organelle targeted by many viruses, which aim to exploit the mitochondrial functions for their benefit. The interaction between the virus and host cell mitochondria eventually determines the outcome of viral infections. The decline in mitochondrial functions and homeostasis is associated with numerous human pathologies including cancer, autoimmune disorders, degenerative neurological disorders, cardiovascular problems, and aging. In a recently published article from our lab, we comprehensively describe the mitochondria-centric events and their significance during dengue virus infection [1]. The liver is a primary organ affected by the dengue virus and acute hepatic manifestations are considered as a warning sign by WHO for progression into severe dengue. Previous studies also signify hepatic involvement and severe mitochondrial injury during dengue infection based on histological examination of liver tissue obtained from lethally ill dengue patients. Our observations suggest that liver cells are highly susceptible to dengue infection and may serve as sites of high viral replication thereby contributing to viremia. We observed copious levels of mitochondrial injury in dengue-infected liver cells with a marked increase in highly swollen mitochondria during the course of infection.

When subjected to physiological stresses or insults, mitochondria stage a graded response involving morphological alterations through fission and fusion events, resulting in the segregation of damaged mitochondria and their subsequent elimination through mitochondrial-selective autophagy or mitophagy. This aspect of mitochondrial quality control is orchestrated in a highly regulated fashion by mitochondrial fusion and fission proteins, and mitophagy mediators. By extensive biochemical and microscopy analysis with fluorescent reporters to analyze macroautophagy/autophagy and mitophagy flux we observed that dengue-triggers global autophagy but specifically inhibits mitophagy. Further investigations revealed that dengue derails the well-established pathway of flagging damaged mitochondria for elimination through mitophagy by downregulating the expression of PINK1 and PRKN/parkin, two proteins that play a crucial role in the flagging process. The mitophagy flux assays also suggest that dengue inhibits other modes (ubiquitin independent) of mitophagy, in addition to the inhibition of PINK1-PRKN-dependent (ubiquitin dependent) mitophagy. The defect in mitochondrial quality control in the dengue-infected cells results in the accumulation of damaged mitochondria leading to cellular injury, and death by necrosis. Rapid replenishment of new mitochondria in conjunction with the clearance by mitophagy is required to maintain functional mitochondrial capacity. Paradoxically we observe that dengue prevents mitochondrial biogenesis by downregulating the PPARGC1A/PGC1α-NFE2L2/NRF2 axis of mitochondrial biogenesis, leading to reduced mitochondrial turnover in dengue-infected liver cells.

Previous studies have demonstrated inflammasome activation and release of pro-inflammatory cytokines from dengue-infected cells, but the molecular triggers that drive this phenotype remained elusive. Accumulation of damaged mitochondria is linked to the release of mitochondrial danger/damage-associated molecular patterns such as mt-DNA, mt-lipids, ROS, ATP, etc. In agreement with this fact, we observed a gradual increase in the mt-DNA levels in the cytoplasm and extracellular medium of dengue-infected cells during the course of infection. Due to shared common ancestry with bacteria, mt-DNA is a potent danger signal and triggers the activation of immune cells and upregulation of a pro-inflammatory response. Exposure of naïve monocytes or macrophages to the culture supernatants from DENV-infected hepatic cells is sufficient to trigger NLRP3 inflammasome activation and release of pro-inflammatory cytokines. Hence, maintaining mitochondrial integrity is pivotal for the well-being of organisms because the inherent innate immune mechanisms can interact with the released mitochondrial components in circulation and cause systemic inflammation. Mt-DNA is implicated in several acute and chronic human diseases due to its role as a vital driver of inflammation. PINK1 and PRKN deficiency has also been linked to the release of mt-DNA, enhanced mitochondrial antigen presentation, inflammation, and neurodegeneration.

This study highlights how interactions between the cells of epithelial and myeloid lineage play a central role in propagating inflammatory signaling due to the cellular injury and DAMPs released from virus-affected epithelial cells (Figure 1). Overall this work adds to our understanding that inflammation during DENV infection may be mediated by mt-DNA release as a consequence of disruption in mitochondrial homeostasis and quality control. It also highlights the use of cell-free mt-DNA levels as makers for the prediction of severe dengue disease and impels us to consider chronic liver co-morbidities while evaluating the risk for the development of severe dengue. Mitochondria-based therapeutic strategies are being considered an attractive avenue for the development of therapeutics against many human pathologies associated with aseptic inflammation driven by mitochondrial DAMPs. Similar strategies can be adopted for curbing viral infection-associated hyperinflammation arising due to underlying mitochondria-centric events. Many viruses promote inflammasome activation through cellular DAMPs. For instance, mitochondrial dysfunction and mt-DNA-triggered inflammatory signaling is implicated in SARS-CoV-2-triggered hyperinflammation and acute respiratory syndrome. Hence, the therapeutics that address the underlying reasons for inflammation will have a pan-viral potential in curbing viral infection-triggered inflammation and immunopathogenesis.

**Figure 1.**
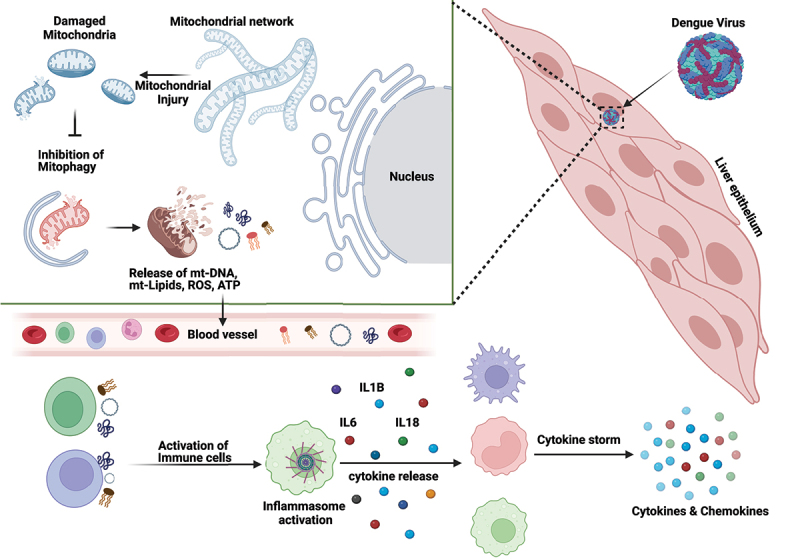
Schematic representation of molecular mechanisms underlying dengue disease pathogenesis. Dengue infection of liver epithelial cells leads to severe mitochondrial injury resulting in mitochondrial dysfunction and damage. Due to the defect in mitophagy in the dengue-infected cell, the damaged mitochondria accumulate resulting in the release of mitochondrial danger/damage-associated molecular patterns/mt-DAMPS such as mt-DNA, mt-lipids, ROS, ATP, etc. These mt-DAMPs may enter the circulation and activate the immune cells triggering inflammation. Activated immune cells then release pro-inflammatory cytokines that further activate naïve bystander cells eventually causing a cytokine storm and dengue disease pathogenesis.

## References

[1] Singh B, Avula K, Sufi SA, Parwin N, Das S, Alam MF, Samantaray S, Bankapalli L, Rani A, Poornima K, Prusty B, Mallick TP, Shaw SK, Dodia H, Kabi S, Pagad TT, Mohanty S, Syed GH. Defective Mitochondrial Quality Control during Dengue Infection Contributes to Disease Pathogenesis. J Virol. 2022 Oct 26;96(20):e0082822. doi: 10.1128/jvi.00828-22. Epub 2022 Oct 5. PMID: 36197108; PMCID: PMC9599662. 36197108 PMC9599662

